# A Decline in CCL3-5 Chemokine Gene Expression during Primary Simian-Human Immunodeficiency Virus Infection

**DOI:** 10.1371/journal.pone.0000726

**Published:** 2007-08-08

**Authors:** Wei Zhao, Bapi Pahar, Juan T. Borda, Xavier Alvarez, Karol Sestak

**Affiliations:** 1 Tulane National Primate Research Center, Covington, Louisiana, United States of America; 2 Tulane University School of Medicine, New Orleans, Louisiana, United States of America; New York University School of Medicine, United States of America

## Abstract

**Background:**

The CC-chemokines CCL3, CCL4 and CCL5 have been found to block the entry of CCR5-tropic HIV into host cells and to suppress the viral replication *in vitro*, but the *in vivo* role of endogenous CC-chemokines in HIV-1 infection is still incompletely understood.

**Methodology/Principle Findings:**

In this study, the primate host CCL3, CCL4 and CCL5 gene expression was evaluated in response to simian-human immunodeficiency virus (SHIV) infection in rhesus macaque model. Five rhesus macaques were inoculated with CCR5-tropic SHIV_SF162P4_. The mRNA levels of CCL3, CCL4 and CCL5 were measured by real-time PCR at post inoculation day (PID) 0, 7, 14, 21, 35, 56 and 180 in peripheral blood. In addition, a selected subset of samples from CXCR4-tropic SHIV_Ku1_-infected macaques was included with objective to compare the differences in CC-chemokine down-regulation caused by the two SHIVs. Gut-associated lymphoid tissues (GALT) collected from SHIV_SF162P4_-infected animals were also tested by flow cytometry and confocal microscopy to corroborate the gene expression results. Predictably, higher viral loads and CD4+ T cell losses were observed at PID 14 in macaques infected with SHIV_Ku1_ than with SHIV_SF162P4_. A decline in CC-chemokine gene expression was also found during primary (PID 7-21), but not chronic (PID 180) stage of infection.

**Conclusions:**

It was determined that A) SHIV_SF162P4_ down-regulated the CC-chemokine gene expression during acute stage of infection to a greater extent (p<0.05) than SHIV_Ku1_, and B) such down-regulation was not paralleled with the CD4+ T cell depletion. Evaluation of CC-chemokine enhancing immunomodulators such as synthetic CpG-oligonucleotides could be explored in future HIV vaccine studies.

## Introduction

Chemokines play a critical role in the host defense against viruses by mobilizing leukocytes to sites of infection, and they also play a homeostatic role in secondary lymphoid tissues [1]. Structurally, chemokines are divided into the C, CC, CXC, and CX_3_C subclasses on the basis of their N-terminal cysteine residues spacing [2]. The majority of known chemokines belong to CC or CXC category [2]. Since CC chemokine receptor 5 (CCR5) and CXC chemokine receptor 4 (CXCR4) were identified as major co-receptors for HIV-1 entry into target cells, it was suggested that these chemokine receptors and their ligands are involved in the transmission and replication of HIV-1 [3], [4]. HIV-1 isolates that use CCR5 as co-receptor (R5 viruses) are predominantly transmitted and persist throughout the infection. By contrast, HIV-1 isolates that use CXCR4 (X4 viruses) commonly emerge in infected individuals at later stages of infection [3]. The importance of CCR5 for HIV-1 transmission was underscored by observation that certain individuals who had been repeatedly exposed to HIV-1 but remained uninfected had a defect in CCR5 expression [5]. It has been documented that mucosa-associated CD4+ T cells, macrophages, and dendritic cells express CCR5 which makes them prime targets for the virus [6]–[8].

Members of the CC-chemokine family, such as macrophage inflammatory protein (MIP)-1α (CCL3), MIP-1β (CCL4) and RANTES (CCL5) are the natural ligands for CCR5. They have been shown to inhibit HIV-1 entry into host cells *in vitro* by competing with viral Env protein for binding [9]–[11], and by down-regulating CCR5 surface expression [12]. However, the *in vivo* role of endogenous CC-chemokines in the pathogenesis of HIV-1 infection is still incompletely understood. A number of studies have analyzed levels of CCR5 ligands in the HIV-1/SIV-infected individuals or monkeys with conflicting results [13]–[18]. Some of these studies concluded that CCR5 ligands may exert protective role in HIV-1/SIV infection [13], [16]–[18], while others suggested an association between elevated levels of CCR5 ligands and HIV disease progression [14], [15]. Most of these reports were based on data that correspond to chronic stages of HIV-1/SIV infection, while the relationship between host CC-chemokine expression and HIV-1 acute infection still needs to be studied.

In this study, we evaluated the host *in vivo* CCL3, CCL4 and CCL5 gene expression in response to CCR5-tropic SHIV infection. We utilized SHIV_SF162P4_/rhesus macaque model and focused predominantly on acute stage of infection to measure the CC-chemokine gene expression changes in peripheral blood and GALT. In addition, we included a selected subset of samples from SHIV_Ku1_-infected macaques in order to compare the differences between the extent of CC-chemokine down-regulation caused by CCR5-tropic SHIV_SF162P4 _and more pathogenic CXCR4-tropic SHIV_Ku1_ at the acute stage of infection.

## Results

### Viral loads and CD4+ T cell counts in peripheral blood of SHIV_SF162P4_-infected macaques

**Figure 1:**
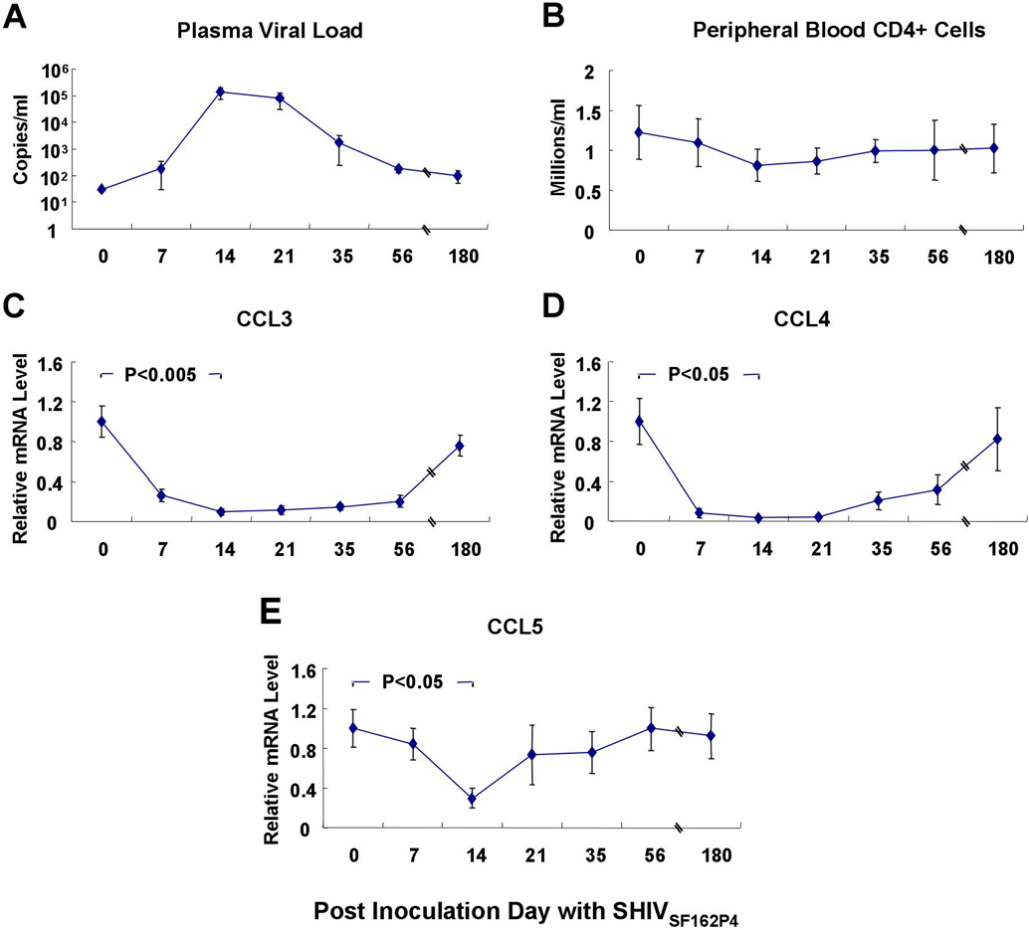
SHIV_SF162P4_ loads in plasma (A), CD4+ T cell counts in peripheral blood (B), relative CCL3 (C), CCL4 (D) and CCL5 (E) gene expression levels in PBMCs at PID 0-180 with SHIV_SF162P4_. Each point on the graph represents the mean fold change in gene expression relative to pre-infection level±SE (n = 5).

All five SHIV_SF162P4_-inoculated macaques became infected as determined by individual viral load measurements. Highest viral loads were detected at PID 14 with an average of ∼10^5^ of viral copies (Fig. 1A). The lowest average peripheral CD4+ T cell counts were detected between PID 14 and 21 (Fig. 1B).

### Down-regulation of CC-chemokine gene expression in peripheral blood of SHIV_SF162P4_-infected macaques

The changes of CCL3, CCL4 and CCL5 gene expression levels were studied in peripheral blood over the period of half year following the SHIV_SF162P4_ inoculation. Decreased expression of all three CC-chemokines was found upon infection. The lowest gene expression was detected at PID 14, coinciding with the peak of primary SHIV_SF162P4_ infection (Fig. 1A, C, D and E). By PID 14, CCL3, CCL4 and CCL5 mRNA levels decreased 5.9–13.6, 3.5–27.5, and 2.3–5.4-folds, respectively. These levels were significantly lower (p<0.005 for CCL3; p<0.05 for CCL4 and CCL5) than those prior to infection (Fig. 1C, D and E). However, such a decline of CC-chemokine gene expression occurred only temporarily, because starting from PID 21 and at later time points these gene expression values showed relative increase. By PID 180, expressions of all three chemokine genes were no longer significantly different from those prior to infection.

### Down-regulation of CC-chemokine gene expression in GALT of SHIV_SF162P4_-infected macaques

**Figure 2:**
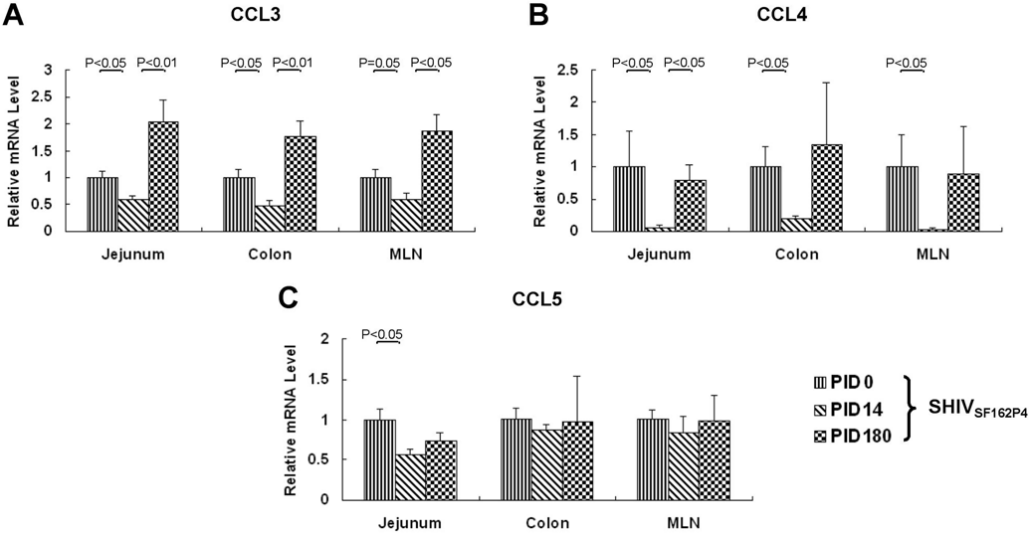
Relative CCL3 (A), CCL4 (B) and CCL5 (C) expression levels in GALT tissues at different stages of SHIV_SF162P4_ infection (n = 5). PID 0 was used as the calibrator (value = 1).

To determine whether the decline of CC-chemokine gene expression occurred also in GALT of SHIV_SF162P4_-infected macaques, biopsy samples of colon, jejunum and MLN were obtained at PID 0, 14 and 180. At PID 14, CCL3 and CCL4 mRNA levels in colon, jejunum and MLN were lower (p≤0.05) than those at PID 0 (Fig. 2A and B). CCL5 mRNA decreased in jejunum (p<0.05) but not in colon and MLN. In agreement with results generated with PBMC, down-regulation of CC-chemokines occurred also in GALT at PID 14. By PID 180, expression of CC-chemokines returned to pre-infection levels (CCL4 and CCL5), or even increased (CCL3).

### *In situ* enumeration of CCL4+ cells in SHIV_SF162P4_-infected macaques

**Figure 3:**
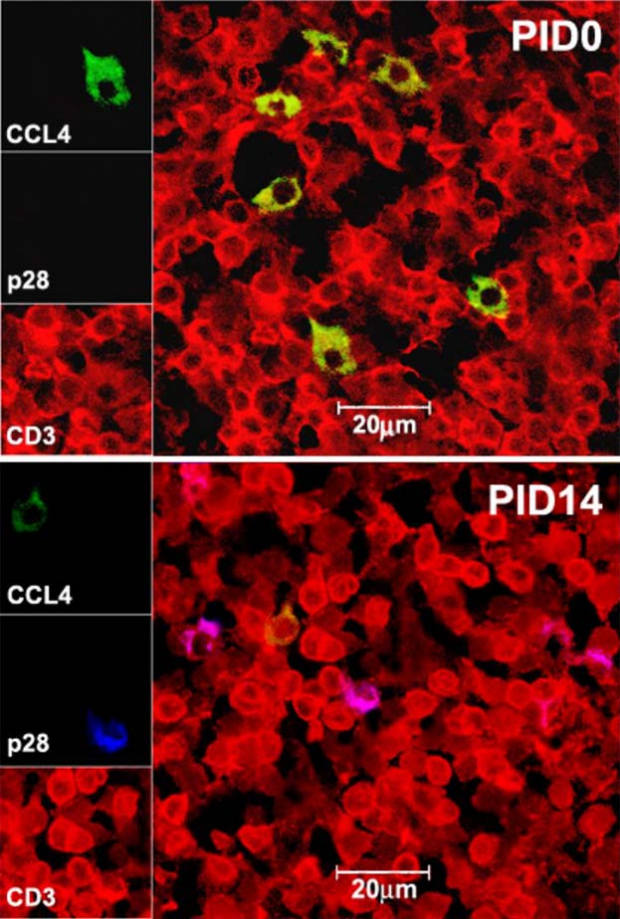
Triple-label confocal microscopy of CCL4+ cells in mesenteric lymph nodes obtained from control (PID 0) and SHIV_SF162P4_-inoculated macaques at PID 14. T lymphocytes (CD3+) are red, CCL4+ cells are green, SHIV_SF162P4_-infected cells (p28+) are blue. Overlap of red and green fluorescence is seen as yellow, overlap of red and blue as pink. Higher number of CD3+CCL4+ cells was seen at PID 0 than at PID 14 (Table 1) while CD3+p28+ cells could be seen at PID 14 but not at PID 0 indicating the successful spread of virus into GALTs following mucosal inoculation.

**Figure 4:**
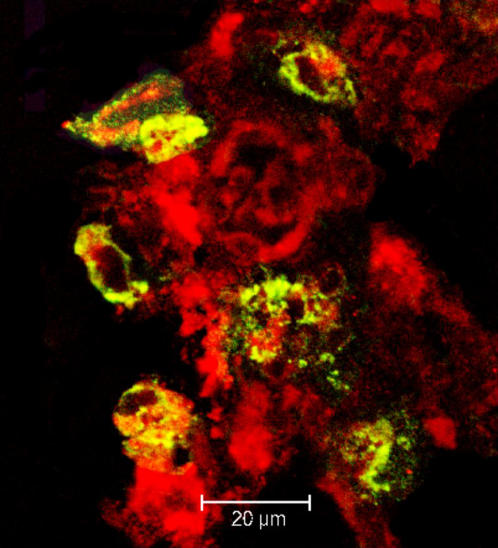
Macrophages were also identified as CCL4+ cells. Macrophages (CD68+) are red, CCL4+ cells are green, overlap of red and green fluorescence is seen as yellow.

To corroborate the mRNA results, and to determine the origin and number of CC-chemokine-producing cells in SHIV_SF162P4_-infected macaques, confocal microscopy and MLN tissues were used. The CCL4 was selected as the CC-chemokine representative. The T lymphocytes (CD3+), macrophages (CD68+), and CCL4+ cells were identified directly *in situ* by tri-color staining (Figs. 3 and 4). Higher number of CD3+CCL4+ cells was seen at PID 0 than at PID 14 (Table 1) while CD3+p28+ cells were seen at PID 14 but not at PID 0 indicating the rapid spread of virus into GALTs following mucosal inoculation (Fig. 3). In addition to CD3+ cells, CD68+ macrophages were also identified as CCL4+ cells (Fig. 4). In agreement with real-time PCR data, the counts of CD3+CCL4+ and CD68+CCL4+ cells were lower (p<0.05) at PID 14 than they were at PID 0 (Table 1).

**Table 1:** Absolute (counts) and relative (%) levels of CCL4+ cells in MLN following SHIV_SF162P4_ inoculation

Sample Date	Animal	CCL4+CD3+ (count)^a^	% CCL4+CD3+ over total CD3+	CCL4+CD68+ (count)^a^	% CCL4+CD68+ over total CD68+
PID 0	Animal 1	12	0.89	7	1.92
	Animal 2	7	0.76	8	2.24
	Animal 3	8	0.78	6	2.11
	Mean±SEM	9±2	0.81±0.04	7±1	2.09±0.09
PID 14	Animal 1	4	0.65	3	0.82
	Animal 2	2	0.26	4	1.12
	Animal 3	3	0.55	3	1.03
	Mean±SEM	3±1	0.49±0.12	3±1	0.99±0.09

a Number of cells/0.5 mm^2^ section.

### Flow cytometry of CCL4+ cells in peripheral blood of SHIV_SF162P4_–infected macaques

**Figure 5:**
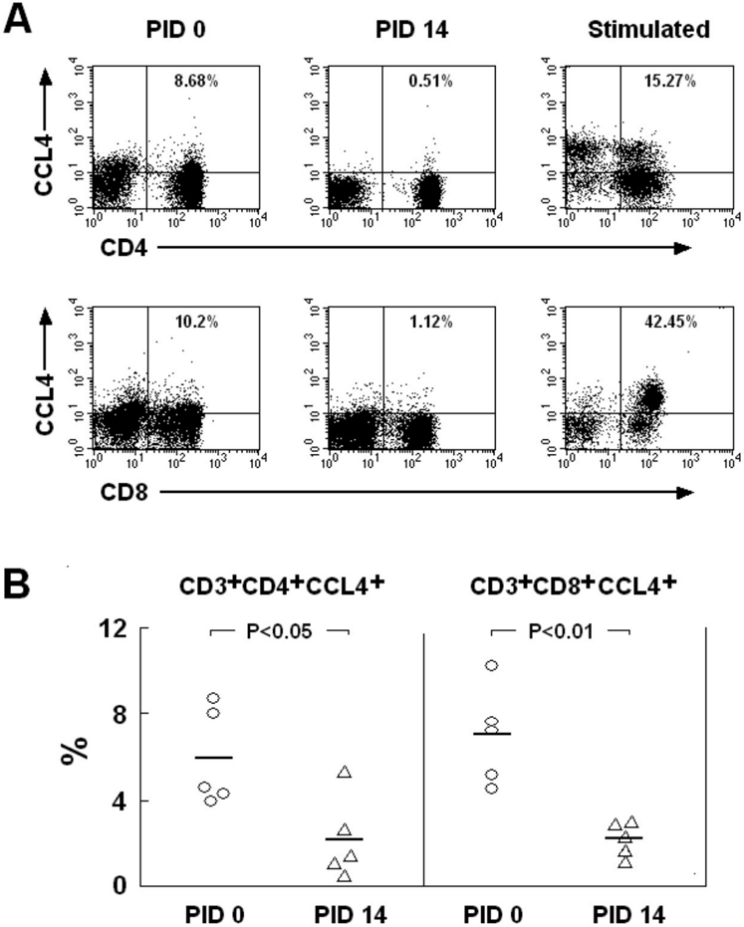
The proportions of peripheral blood CD3+CD4+CCL4+ and CD3+CD8+CCL4+ T lymphocytes were compared at PID 0 and PID 14 with SHIV_SF162P4_ by flow cytometry (A). Gating was performed via CD3+ lymphocyte population. Statistically significant differences between CD3+CD4+CCL4+ and CD3+CD8+CCL4+ cells over CD3+ lymphocytes at PID 0 vs. PID 14 (n = 5) are shown (B).

Four-color flow cytometry was used to corroborate the gene expression results. At PID 0, the average proportions of CD3+CD4+CCL4+ and CD3+CD8+CCL4+ cells over CD3+ T cells were 5.9% and 6.9%, respectively (Fig. 5B). By PID 14, both of these proportions decreased (p<0.05 and p<0.01) to 2.2% and 2.3% (Fig. 5B). In order to be able to differentiate between CCL4+ and CCL4- cells, mitogen-stimulated cells were included as controls (Fig. 5A).

### The extent of CC-chemokine gene down-regulation in blood of SHIV-infected macaques is independent from the level of CD4+ T cell depletion

**Figure 6:**
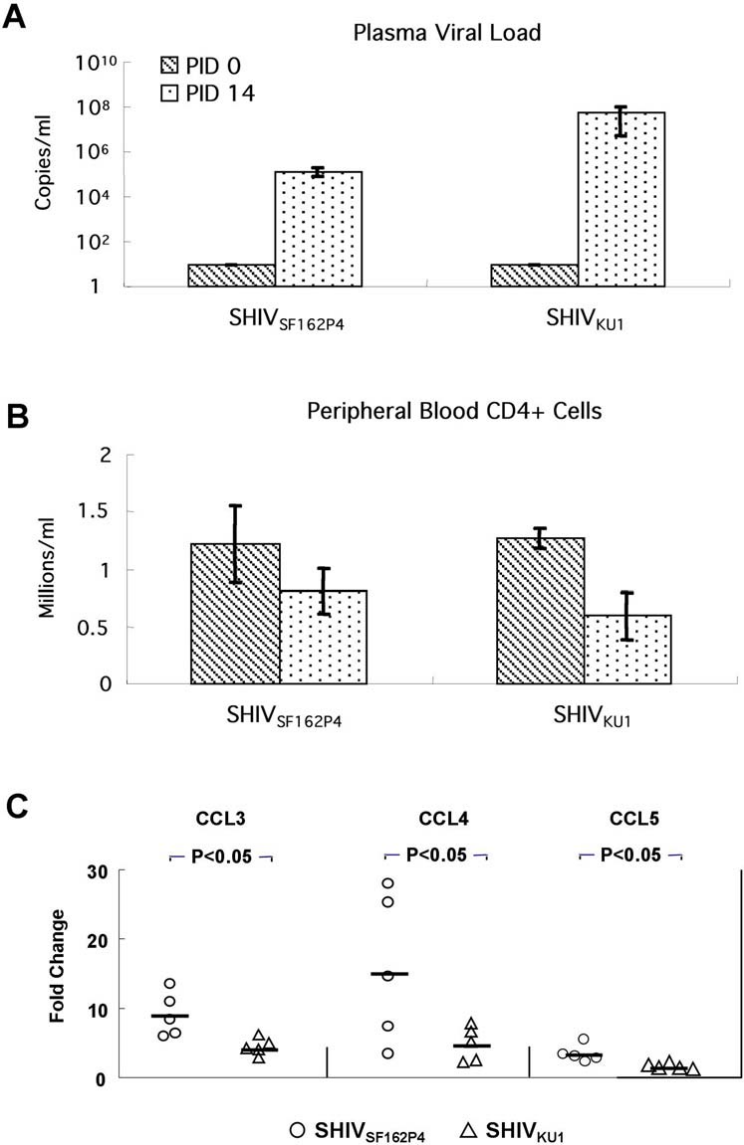
Comparison of SHIV_SF162P4 _and SHIV_Ku1_ primary infection in peripheral blood. Viral loads in plasma (A), CD4+ T cell counts (B), Fold-changes of CCL3, CCL4 and CCL5 gene expression levels in SHIV_SF162P4_- and SHIV_Ku1_–infected macaques at PID 0 vs. PID 14 (C) (n = 5 for each group). Negative sample cut-off for viral load measurements in plasma was <30 copies/ml e.g. PID 0 value.

We hypothesized that SHIV_SF162P4_–induced CC-chemokine down-regulation at acute stage of infection was not proportionately related to peripheral CD4+ T cell depletion as a result of viral target cell destruction. Therefore, we included in this study a subset of samples obtained from SHIV_Ku1_-infected macaques (PID 0 and 14). In accord with previous reports [19], [20], it was confirmed that SHIV_Ku1_ infection was at PID 14 associated with greater plasma viral loads and more profound loss of peripheral CD4+ T cells than that caused by SHIV_SF162P4_ (Fig. 6A and B). Interestingly, SHIV_SF162P4_ caused by PID 14 5.9–13.6, 3.5–27.5, and 2.3–5.4-fold CCL3, CCL4 and CCL5 gene down-regulation respectively, while only 2.8–6.2, 2.3–7.8, and 1.2–2.2-fold down-regulation was measured in SHIV_Ku1_-infected macaques (Fig. 6C). These differences in CC-chemokine down-regulation between SHIV_SF162P4_- and SHIV_Ku1_-infected macaques were significant (p<0.05).

## Discussion

Main objective of our study was to evaluate the CC-chemokine gene expression changes in SHIV_SF162P4_-infected macaques. We found that at primary/acute stage of infection, CC-chemokine genes were markedly down-regulated, which coincided with the peak of viremia. Gene expression results were consistent with direct measurements of chemokine production–corroborated by confocal microscopy and flow cytometry. Results generated with peripheral blood and GALT samples were also consistent. In agreement with our results, a decline in CCL4 gene expression by CD8+ intraepithelial lymphocytes (IEL) was observed at the acute stage of SIV infection [21]. Study by Wen and colleagues [22] also showed that expression of CCL3, CCL4 and CCL5 decreased in U937 promonocytes in response to HIV-1 infection. In addition, several studies reported up-regulation of CC-chemokines during chronic phase of HIV/SIV infection [14], [15], [23]. These reports are not in conflict with our results. Results shown here indicate however that down-regulation of CCR5 ligands occurred only at primary (PID 14) but not at chronic stage of SHIV_SF164P4_ infection (PID 180). At the chronic stage of SHIV_SF164P4_ infection, expression of the CCR5 ligands returned to pre-infection levels (CCL4 and CCL5), or even increased (CCL3) in all three GALTs.

There are several possible mechanisms involved in decline of CC-chemokines during primary SHIV infection. Since lymphocytes are CC-chemokine producing cells, the depletion of CD4+ T lymphocytes could be one of the reasons. Based on measurable and significant CC-chemokine gene down-regulation caused by SHIV_SF162P4_ that is known to be less pathogenic in term of its ability to deplete the peripheral CD4+ T cells than other SHIVs, SIV or HIV, a subset of samples obtained from SHIV_Ku1_-infected macaques (PID 0 and 14) was included in this study. It was hypothesized that SHIV_SF162P4_–induced CC-chemokine down-regulation is not directly related to peripheral CD4+ T cell depletion-as a result of viral target cell destruction. This hypothesis was conclusively corroborated by measurements of CC-chemokine gene expression at PID 14 when SHIV_SF162P4_ was found to cause significantly greater gene down-regulation than SHIV_Ku1_. Such result suggests that decline of CC-chemokines cannot be solely attributed to CD4+ T cell depletion, but it is more complex and likely linked to other pathways triggered by acute SHIV infection. One of them might be related to SHIV gene products, which modulate transcriptional levels of host response genes [24]. For example, HIV accessory gene product Vpr has been reported to suppress the host CC-chemokine gene expression [25]. Another candidate pathway is the interaction between the HIV/SIV gp120 and the CCR5 chemokine co-receptors, which trigger not only viral entry but also other signal transduction cascades that are impacting host CC-chemokine gene expression [26], [27]. Elucidating such pathways in context of CC-chemokine production can be subject of future studies.

CCL3, CCL4 and CCL5 function as natural ligands for CCR5, the major HIV/SIV co-receptor [3]. CC-chemokines are known to inhibit the CCR5-mediated HIV/SIV infection by blocking the viral entry into host cells [9], [10]. In addition, CC-chemokines play a role in directing the cell movements necessary for the initiation of T cell immune responses [1], co-stimulate the T cell proliferation, and augment the cytolytic capacity of T and NK cells [28], [29]. Thus, suppression of CC-chemokines during primary/acute stage of infection may be an event that facilitates the evasion of HIV/SIV from the host immune response. It was demonstrated by several authors that production of CC-chemokines was associated with vaccine-mediated protection from SIV challenge [13], [16], [30]. Furthermore, systemic therapy with CC-chemokine homologues decreased SIV_mac251_ replication in rhesus monkeys [31]. These reports are in agreement with our findings by corroborating the notion that CC-chemokine down-regulation may facilitate virus spread during acute stage of infection.

It may be unexpected that CCL3, CCL4 and CCL5 are down-regulated at the acute phase of SHIV infection, since most of the CC-chemokines belong to proinflammatory chemokines and are generally considered to be produced only in response to pathological conditions [32]. However, the constitutive expression of CCL3, CCL4 and CCL5 was detected in monocytes [33] and CD8+ intraepithelial lymphocytes [21]. In this study, highly sensitive real-time PCR was used to measure the expression of the above CC-chemokines in PBMC and GALTs before and after SHIV_SF162P4_ infection, and it was concluded that these chemokines are down-regulated during primary infection.

In summary, we hypothesize that an interventional up-regulation of CC-chemokines might lead to better outcome of HIV/SIV primary infection. Evaluation of CC-chemokine enhancing immunomodulators such as synthetic CpG-oligonucleotides could be explored in future SIV/HIV vaccine studies.

## Materials and methods

### Animals and virus

Five adult rhesus macaques (*Macaca mulatta*) of Indian origin were inoculated with 5,000 TCID_50_ of CCR5-tropic SHIV_SF162P4_ via intrarectal route [19]. SHIV_SF162P4_ was obtained from the Simian Retrovirus Core Laboratory at the TNPRC (Tulane National Primate Research Center). Animals were housed at the TNPRC under biosafety level 2 conditions in accordance with the regulations of the American Association for Assessment and Accreditation of Laboratory Animal Care standards. In addition, five adult rhesus macaques of Indian origin were inoculated with CXCR4-tropic SHIV_Ku1_ by using the same route and dose as described above for SHIV_SF162P4_. SHIV_Ku1_ was obtained from NIH (www.aidsreagent.org). Both SHIVs were expanded and titrated *in vitro* as described elsewhere [34], [35].

### Sample collection and storage

From the 5 animals inoculated with SHIV_SF162P4_, EDTA blood was collected prior to inoculation (PID 0), and at PID 7, 14, 21, 35, 56 and 180. GALT samples (jejunum, colon and MLN) were collected via laparotomy biopsies at PID 0, 14 and 180. The peripheral blood mononuclear cells (PBMC) were isolated from EDTA blood using the lymphocyte separation medium (Cappel, Aurora, OH) according to instructions of manufacturer, and stored in lysis buffer (Qiagen, Valencia, CA) (10×10^6^ cells/600 µl of lysis buffer) at −80°C until RNA isolation. The GALT samples were cut into 20–30 mg pieces, placed in 5 volumes of RNAlater (Ambion, Austin, TX), incubated at 4°C overnight and stored at −20°C until RNA extraction. From the 5 animals inoculated with SHIV_Ku1_, samples of EDTA blood were obtained at PID 0 and 14 in order to compare measurements of a) plasma viral load, b) peripheral CD4+ T cell counts and c) CC-chemokine gene expression between SHIV_SF162P4_- and SHIV_Ku1_–infected macaques.

### RNA isolation and cDNA preparation

Total RNA was isolated from 20–30 mg of GALT tissue or 10×10^6^ of PBMC using the RNeasy mini kit (Qiagen, Valencia, CA). Prior to RNA isolation, tissue was homogenized by 3 pulses of sonication, each at 60 W for 3 sec. All samples were treated with RNase-free DNase (Qiagen). cDNA was prepared using the random hexamer primers (Integrated DNA Technologies, Skokie, IL) and M-MLV reverse transcriptase (Promega, Madison, WI) according to instructions of manufacturer.

### Evaluation of chemokine gene expression by real-time PCR

Real-time PCR was carried out using the ABI Prism 7700 Sequence Detection System (Applied Biosystem, Foster City, CA). Each PCR reaction included 12.5 µl of master mix (DyNAmo HS SYBR Green qPCR Kit, MJ research, Waltham, MA). It contained modified DNA polymerase, SYBR Green I, 5 mM MgCl_2_, dNTP mix, 0.5 µM of forward and reverse primers, 1×ROX reference dye, and 100 ng of the first strand cDNA in deionized water. After heating each sample to 95°C for 15 min, 40 cycles of 94°C for 10 sec, 55°C for 30 sec, and 72°C for 30 sec followed. After the thermal cycles, melting curves of the amplicons were generated by heating the reaction mixture to 95°C for 15 sec, then to 60°C for 20 sec, and slowly increasing the temperature to 95°C over a period of 20 min. The fluorescence was measured as a function of temperature. All samples were run in duplicates and the PCRs for the housekeeping glyceraldehyde-3-phosphate dehydrogenase (GAPDH) and the target genes (Table 2) were run in parallel for each sample. PCR reactions were carried out on a 96-well plate (Applied Biosystems, Foster City, CA) using the 25 µl/reaction volumes. Results were analyzed by SDS 1.9.1 Software (Applied Biosystems). The melting curves were obtained with ABI Prism 7700 software, version 1.7(http://www.appliedbiosystems.com/support/software/7700pdates.cfm, ABI). The specificity of real-time PCR was verified by melting curve analysis. Housekeeping gene GAPDH was used to normalize the RNA in tested samples. The variation in GAPDH Ct value among the RNA samples was ≤3%. 2^−ΔΔCt^ method [36] was used in calculating the fold-relationships in gene expression between the time points. Pre-infection (PID 0) was used as calibrator to generate the graphs shown in Fig 1 and 2.

**Table 2:** Rhesus macaque-specific primers for real-time PCR

Gene	GenBank #	Sequences (5′-3′)	Start-End Position	Amplicon Length (bp)
CCL3	AF449266	F: CAACATTTGCTGCTGACACC	68–87	103
		R: CACTGGCTGTTGGTCTCAAA	151–170	
CCL4	AF449267	F: CCAGACCAAAAGAGGCAAGC	195–214	76
		R: TTCCAGGTCATTAACATACTCC	249–270	
CCL5	AF449268	F: TACACCAGTGGCAAGTGCTC	154–173	100
		R: TGTACTCCCGAACCCATTTC	234–253	
GAPDH	CO774505	F: GGGAGCCAAAAGGGTCATCA	378–397	91
		R: GAGGCTGTTGTCATACTTCTC	448–468	

### Evaluation of viral loads by real-time PCR

Quantitative assessment of viral RNA load in plasma samples obtained from peripheral blood of SHIV-inoculated macaques was determined by real-time reverse transcriptase PCR as described in detail elsewhere [37]. The virus used in this study was accurately and equivalently quantified in this assay. The values were expressed as viral RNA copies per ml of plasma.

### Enumeration of CCL4-producing cells in MLN by confocal microscopy

MLN biopsies obtained from 3 animals prior to infection (PID 0) and at PID 14 were cryosectioned to 10–16 µm and subjected to immunofluorescence staining as previously described [38]. Briefly, the tissues were blocked with 10% donkey serum (Sigma, St. Louis, MO) in PBS containing 0.2% fish skin gelatin (Sigma, St. Louis, MO). The sections were subsequently stained with primary antibodies to detect CCL4+ cells, SHIV-infected cells, T cells and macrophages (anti-CCL4, SIV gag p28, CD3, and CD68 antibodies were purchased from R&D Systems, Microbix, Dako and BD Pharmingen, respectively). Negative-control samples were stained with matched isotype control antibodies and no positive cells were identified (not shown). Primary staining was followed by incubation with secondary antibodies conjugated to fluorochromes. Images were captured by a Leica TCS SP2 laser-scanning microscope (Leica Microsystems, Exton, PA) and viewed using the Leica imaging software [38].

### Flow cytometry analysis of CCL4 expression in CD3+CD4+/CD8+ T cells

Presence of intracellular CCL4 in PBMC was evaluated by four-color flow cytometry. The following anti-human monoclonal antibodies that cross-react with rhesus macaques were used: Phycoerythrin (PE)-conjugated anti-MIP-1β/CCL4 (clone D21-1351), Fluorescein isothiocyanate (FITC)-conjugated anti-CD3 (clone SP34), Peridinin Chlorophyll (PerCP)-conjugated anti-CD8 (clone SK1), and Allophycocyanin (APC)-conjugated anti-CD4 (clone SK3). All antibodies were purchased from BD-Pharmingen (Pharmingen, San Diego, CA). PBMCs (5×10^5^ per sample) were first incubated with fluorochrome-conjugated antibodies to CD3, CD4 and CD8 at 4°C, in dark, for 30 min. The cells were then washed with PBS containing 0.2% fetal bovine serum (FBS, Invitrogen, Chicago, IL), and permeabilized with 1× fixation/permeabilization solution (BD Biosciences) for 10 min at room temperature. Following two washes with PBS containing 0.2% FBS, the cells were incubated with anti-CCL4 for 30 min at 4°C. After washing, cells were resuspended in 2% paraformaldehyde/PBS. Positive control PBMCs were stimulated with 100 ng/ml of phorbol myristate acetate (PMA, Sigma) and 1 µg/ml of ionomycin (Sigma) for 6 h at 37°C, 5% CO_2_. Brefeldin-A (10 µg/ml, Sigma) was added for final 5 h. Mitogen-stimulated control PBMCs were stained and processed as described above for unstimulated samples. Negative-control samples were stained with matched isotype control antibodies. Stained cells were kept protected from light at 4°C and acquisition was completed within 48 h. Data acquisition was performed using the FACS-Aria Flow Cytometer (BD Biosciences), and collected data were analyzed by Cell Quest software (Becton Dickinson).

### Statistical analysis

CCL3, CCL4 and CCL5 mRNA levels, CCL4+ cell numbers in MLN and percentages of CCL4+ cells in peripheral blood CD4+ and CD8+ T cell populations were compared between selected time points of SHIV_SF162P4_-infected macaques by Student's t test and a p<0.05 was considered statistically significant. In addition, the extent of CC-chemokine down-regulation in peripheral blood of SHIV_SF162P4_- and SHIV_Ku1_-infected macaques was compared at PID 14 by Student's t test (p<0.05).
